# The impact of COVID-19 on the mental health and substance use health (MHSUH) workforce in Canada: a mixed methods study

**DOI:** 10.1186/s12960-023-00797-6

**Published:** 2023-02-08

**Authors:** Christine Tulk, Mary Bartram, Kathleen Leslie, Jelena Atanackovic, Caroline Chamberland-Rowe, Ivy Lynn Bourgeault

**Affiliations:** 1 Department of Psychology, Carleton University, 1125 Colonel By Drive, Ottawa, ON K1S 5B6 Canada; 2 Mental Health Commission of Canada, 350 Albert Street, Suite 1210, Ottawa, ON K1R 1A4 Canada; 3 Faculty of Health Disciplines, Athabasca University, 1 University Drive, Athabasca, AB T9S 3A3 Canada; 4 School of Sociological and Anthropological Studies, University of Ottawa, 120 University Private, Ottawa, ON K1N 6N5 Canada; 5 Nova Scotia Health, 90 Lovett Lake Court, Halifax, NS B3S1B8 Canada

**Keywords:** Mental health, Substance use health, Workforce capacity, COVID-19

## Abstract

**Background:**

The increased need for mental health and substance use health (MHSUH) services during the COVID-19 pandemic underscores the need to better understand workforce capacity. This study aimed to examine the pandemic’s impact on the capacity of MHSUH service providers and to understand reasons contributing to changes in availability or ability to provide services.

**Methods:**

We conducted a mixed method study including a pan-Canadian survey of 2177 providers of MHSUH services and semi-structured interviews with 13 key informants. Survey participants answered questions about how the pandemic had changed their capacity to provide services, reasons for changes in capacity, and how their practice had during the pandemic. Thematic analysis of key informant interviews was conducted to gain a deeper understanding of the impact of the pandemic on the MHSUH workforce.

**Results:**

Analyses of the survey data indicated that the pandemic has had diverse effects on the capacity of MHSUH workers to provide services: 43% indicated decreased, 24% indicated no change, and 33% indicated increased capacity. Logistic regression analyses showed that privately funded participants had 3.2 times greater odds of increased capacity (*B* = 1.17, *p* < 0.001), and participants receiving funding from a mix of public and private sources had 2.4 times greater odds of increased capacity (*B* = 0.88, *p* < 0.001) compared to publicly funded participants. Top reasons for decreases included lockdown measures and clients lacking access or comfort with virtual care. Top reasons for increases included using virtual care and more people having problems relevant to the participant's skills. Three themes were constructed from thematic analysis of key informant interviews: the differential impact of public health measures, long-term effects of pandemic work conditions, and critical gaps in MHSUH workforce data.

**Conclusions:**

The COVID-19 pandemic has had a substantial impact on the capacity of the MHSUH workforce to provide services. Findings indicate the importance of increasing and harmonizing funding for MHSUH services across the public and private sectors, developing standardized datasets describing the MHSUH workforce, and prioritizing equity across the spectrum of MHSUH services.

**Supplementary Information:**

The online version contains supplementary material available at 10.1186/s12960-023-00797-6.

## Background

The COVID-19 pandemic has changed nearly every aspect of daily life for Canadians, with increases in stressors such as social isolation, financial stress, disrupted childcare, and worries about COVID-19 infection becoming part of a “new normal”. Early evidence indicates a serious impact on mental health and substance use health (MHSUH), with notable increases in depressive symptoms and higher use of alcohol and cannabis, combined with difficulties accessing treatment services [1]. The MHSUH workforce is the backbone of the response to this increased need for MHSUH services; however, data on the availability and abilities (i.e., capacity) of this workforce are lacking, and there has been little research attention towards issues faced by individuals working to provide these services [2].

Early evidence indicates that most health care systems around the world were unprepared and lacked the necessary resources to respond to the surge in demand for MHSUH services [3]. Public health measures implemented to reduce viral transmission have resulted in widespread disruptions to critical MHSUH services [4] along with decreased activity in some areas (e.g., in-patient admissions, new referrals, psychological assessments) and increased activity in other areas (e.g., crisis intervention, virtual services, counselling services for other health care workers) [5–8]. Many healthcare resources including MHSUH staff were diverted for care of COVID-19 patients and preventive health services, such as screening and vaccination [9, 10]. Physical distancing protocols have limited in-person care and caused a consequent rapid shift to virtual care, raising both technical and equity considerations [11–14].

These challenges have been uniquely felt in Canada where MHSUH services are not universally covered by the publicly funded health care system. Rather, MHSUH services are a precarious mix of publicly insured services provided in hospitals and by physicians, other publicly funded services provided on a limited basis in community and virtual settings, and services provided by practitioners in private practice and private treatment facilities paid for either directly by the client or through employee insurance coverage. MHSUH services provided by the public sector were already underfunded prior to the pandemic [15]. Moreover, private insurance coverage was insufficient in many cases to cover the costs of MHSUH services such that not being able to pay for services was one of the top reasons for not receiving adequate MHSUH care even before the pandemic worsened the financial situation of many Canadians [16].

The disproportionate impact of the pandemic on women, minorities, and Indigenous groups [2] also raises concerns about how the pandemic is affecting MHSUH service providers with these social identities. Although data specific to the MHSUH workforce are limited in Canada, available data indicate that approximately 75% of psychologists and 87% of social workers across Canada are women [17]. Given that seven out of ten of the world’s health workforce are women [18] and that this proportion is even higher in Canada exceeding 80% [19], it is reasonable to presume that the MHSUH workforce is also predominantly women. These women face a double burden of strain not only from longer and more stressful shifts at work, but also additional care work at home as the primary providers of unpaid care work [20], and these effects are compounded for minority and Indigenous groups working to provide culturally and linguistically appropriate services to segments of the population that were already underserviced.

To summarize, Canada’s MHSUH workforce is complex, yet we have little empirical evidence about how the COVID-19 pandemic has affected workforce capacity in the public or private sectors. Given that information on the MHSUH workforce is crucial for effective planning to deliver services and to develop policy to provide equitable access to these services, the first objective of this research was to address this critical knowledge gap by examining (1) changes in practice characteristics of MHSUH service providers across Canada and their capacity to provide services; (2) whether factors such as gender, type of funding and work role were associated with changes in capacity; and (3) potential reasons for increases and decreases in capacity to provide services. The second objective was to examine the broad impact of the pandemic on the MHSUH workforce by exploring issues critical to workforce capacity and planning.

## Methods

### Study design

This study included two components, a cross-sectional survey and key informant interviews, designed to examine issues affecting capacity at the practice level and to explore workforce-level issues that affect capacity more broadly. The survey component assessed the impact of the pandemic on practice characteristics (e.g., work hours, number of clients), changes in capacity to provide services, and reasons for changes in capacity to provide services using a web-based questionnaire administered during the third wave of the COVID-19 pandemic in Canada. To complement the practice-level information gathered using the survey, interviews were conducted with 13 key informants working in positions that offer a broad view of issues affecting workforce capacity and richer detail regarding reasons for changes in workforce capacity during the initial period of lockdown and social distancing measures through subsequent waves of the pandemic. Both the survey questionnaire and interview protocol were developed based on consultations between the research team and a pan-Canadian advisory committee comprising subject matter experts from different sectors of the MHSUH workforce.

### Sampling and data collection

Survey data from 2177 participants working in 23 MHSUH occupations were collected from mid-March 2021 to May 2021 (approximately 12–14 months after the start of lockdown and social distancing measures in Canada) using a web-based questionnaire hosted on the Qualtrics survey platform. Survey participants were recruited by posting recruitment notices on social media platforms and by crowdsourcing using a Canadian market research company to target Francophones working in health care professions. In addition, we used purposive sampling to recruit 13 key informants from relevant stakeholder organizations to participate in interviews conducted in April and May 2021.

### Survey questionnaire

#### Occupational characteristics

Participants were asked to identify their primary MHSUH work role and to indicate if they were licensed, certified, or regulated in this role. They were also asked to indicate how many hours per week and how many clients or patients they typically provided services to in an average week both before and during the pandemic and where these clients or patients resided. There were two slider items asking participants the percentage of services typically provided face-to-face (in person) and virtually (telephone or internet/app/text-based) both before and during the pandemic. Participants were instructed to select the percentages of face-to-face and virtual services so that the total percent was 100.

#### Type of funding

A single item was created for the present study to ask participants how they are typically funded. Type of funding was coded as public if participants indicated receiving only public funding from government sources, private if participants indicated receiving only private funded (i.e., funded through benefits programs, donations or direct payment), or mixed if participants indicated receiving both public and private funding.

#### Impact on capacity

To assess the impact of the pandemic on capacity, we included one item asking if participants’ ability or availability to provide MHSUH services had increased, decreased, or remained unchanged since the start of the pandemic. Participants who indicated an increase were given a checklist of potential reasons for increased capacity, and participants who indicated a decrease were given a checklist of potential reasons for decreased capacity. They were also asked if there were any services that they were capable of but not presently providing. If they indicated they could provide more services, they were given a checklist of items that would enable them to do so. Participants could select multiple items for each checklist.

#### Demographics

Demographics included age, gender identity, and whether the participant identified as racialized, Indigenous, or as a person living with a disability. Participants were also asked to indicate the provinces and territories where their clients resided and whether clients resided in population centers that were large (100,000 or larger), medium (between 30,000 and 99,999), small (between 1000 and 29,999), rural (1000 or less), or remote (regardless of population size).

### Survey results

All statistical analyses for the survey data were conducted using IBM SPSS Statistics Version 27. Analyses began with conducting descriptive statistics on variables representing demographic and occupational characteristics. This included computing means, standard deviations, and ranges for continuous variables and frequencies for categorical variables. The data were also visualized using histograms, scatterplots, and bar charts. Chi-squared tests, Pearson correlations, and *t-*tests were run to examine bivariate associations between variables. To verify that assumptions for multinomial logistic regression were met, we tested for multicollinearity between predictor variables, examined the case-to-variable ratio, and conducted Chi-squared goodness-of-fit tests between all pairs of categorical variables.

### Sample characteristics

The sample for the survey included 2177 participants who were currently providing MHSUH services across Canada. Sample characteristics including demographics, primary MHSUH workforce role, and location of patients/clients are shown in Table 1. Language chosen for the survey was 86% (*n* = 1873) English and 14% (*n* = 304) French. Most of the sample (52%) received only public funding from government; however, a substantial percentage (22%) received payment only from private sources including donations, endowments, and direct payment from clients, and an additional 26% were funded through a combination of these private sources and public funding by government.

**Table 1:** Sample characteristics for survey participants

Sample characteristic	*n* (%)
Demographics (*n* = 1510)	
Woman	1214 (80%)
Man	241 (16%)
Non-binary	25 (2%)
Racialized	123 (8%)
Indigenous	64 (4%)
Living with a disability	132 (9%)
Age, mean years (SD in years)	45.54 (13.17)
Primary MHSUH Workforce Role (*n* = 2177)	
Regulated MHT^a,b^	423 (19.4%)
RSW	421 (19.3%)
Nurse^e^	274 (12.6%)
Unregulated MHT^a,b^	219 (10.1%)
Other Role (self-identified)	130 (6.0%)
Addiction counsellor	139 (6.4%)
Psychologist or psychological associate	86 (4.0%)
Case manager	74 (3.4%)
Occupational therapist	62 (2.8%)
Physician (family, psychiatrist, geriatrician, pediatrician, other)	58 (2.7%)
Personal support worker	54 (2.5%)
Peer support/counsellor	49 (2.3%)
Trainee	38 (1.7%)
Crisis counsellor	37 (1.7%)
MH promotion worker	35 (1.6%)
Residential care worker	19 (0.9%)
Housing support worker	17 (0.8%)
Psychosocial rehabilitation worker	16 (0.7%)
Harm reduction provider	13 (0.6%)
Psychoeducator	13 (0.6%)
Location of patients/clients by province/territory (*n* = 2169)	
Ontario	881 (40.8%)
Quebec	308 (14.3%)
British Columbia	297 (13.7%)
Alberta	257 (11.9%)
Manitoba	228 (10.6%)
Saskatchewan	96 (4.4%)
Nova Scotia	94 (4.3%)
Newfoundland and Labrador	71 (3.3%)
New Brunswick	68 (3.1%)
Across Canada	31 (1.4%)
Yukon	18 (0.8%)
Northwest Territories	16 (0.7%)
Nunavut	15 (0.7%)
Prince Edward Island	10 (0.5%)
Location of patients/clients by type of population center (*n* = 1533)	
Large and/or medium population centers	988 (63.6%)
Small, rural, and/or remote	292 (18.8%)
Mix of large/medium and small/rural/remote	273 (17.6%)

MHT = mental health therapist. RSW = registered social worker

^a^Participant selected psychotherapist, clinical counsellor, employee assistance program counsellor, counselling therapist, or other role and provided a description of a counselling occupation not included in the survey response options. ^b^Regulated refers to participants who indicated being a member of a regulatory body. ^c^Participants selected registered nurse, nurse practitioner, clinical nurse specialist, registered/licensed practical nurse, registered psychiatric nurse, or other nurse

### Impact on service modality, number of clients, and hours of work

#### Face-to-face and virtual services

Prior to the pandemic, survey participants indicated that they delivered services face-to-face 88.8% (Mdn = 99%, IQR = 10.0, SD = 20.6%) of the time compared to virtually 11.2% (Mdn = 1%, IQR = 10.0, SD = 20.6%) of the time. This changed during the pandemic, with participants indicating that they delivered services face-to-face only 35.9% (Mdn = 20.5%, IQR = 58.0, SD = 34.9%) of the time compared to virtually 64.1% (Mdn = 79.5%, *IQR* = 58.0, SD = 34.9%) of the time. A paired-samples *t*-test indicated that the increase in virtual services and decrease in face-to-face services were significant, *t*(1661) = 57.59, *d* = 1.41, *p* < 0.001, 95% CI [51.27, 54.89].

#### Number of clients and hours of work

Paired samples *t*-tests were conducted to examine the impact of the pandemic on number of clients and hours per week providing direct services. Results (see Table 2) indicated that survey participants significantly increased both the number of clients per week and number of hours per week directly providing services during the pandemic compared to before the pandemic. Number of clients and hours per week providing direct services by primary MHSUH workforce role and gender identity can be found in Additional file 1.

**Table 2:** Hours and clients per week before and during the pandemic

Variable	Before				During				*ΔM*	*t*	*p*	95% CI	
	*M*	Mdn	IQR	SD	*M*	Mdn	IQR	SD				LL	UL
Hours per week	27.9	30.0	20.0	14.6	28.8	30.0	20.8	14.7	0.8	3.15^a^	0.002	0.3	1.4
Clients per week	22.1	17.0	15.0	38.0	24.0	20.0	20.0	32.8	1.9	2.54^b^	0.01	0.4	3.3

*Notes*. IQR = interquartile range

^a^*n* = 1488, ^b^*n* = 1477

### Impact by gender and funding type

**Fig. 1:**
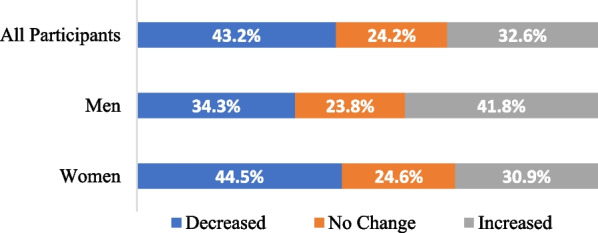
Impact of pandemic on capacity by gender

**Fig. 2:**
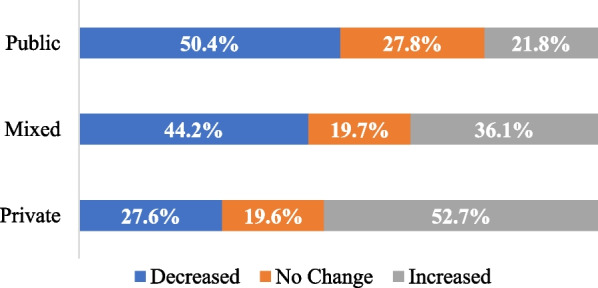
Impact of pandemic on capacity by funding type

Responses to the survey item asking participants if their availability or ability to provide services directly had decreased, not changed, or increased are shown in Fig. 1. A Chi-squared test of independence indicates that responses to this question differed by gender, *χ*^*2*^ (2) = 12.17, *p* = 0.002 (see Fig. 1), and by type of funding, *χ*^*2*^ (4) = 117.07, *p* < 0.001 (see Fig. 2). The number of non-binary, racialized, and Indigenous participants was too few to enable further sub-groups analysis.

We also used multinomial logistic regression to examine the role of gender [man, woman], funding type [private, public, mixed], and primary workforce role [addiction counsellor, unregulated mental health therapist, psychologist, nurse, registered social worker, regulated mental health therapist] on the impact of the pandemic on survey participants' ability and availability to provide services [decreased, no changed, increased]. Results are shown in Table 3. There were no significant differences for odds of decreased capacity versus no change in capacity and no significant differences in odds that men indicated increased capacity compared to women. However, compared to participants receiving only public funding, participants receiving a mix of public and private funding had 2.41 times odds of indicating increased capacity, and participants receiving only private funding had 3.21 times odds of indicating increased capacity. Compared to regulated mental health therapists, psychologists had 0.28 times odds of indicating increased capacity, and nurses had 0.43 times odds of indicating increased capacity. The logistic regression analysis was repeated including geographic location of clients [not remote, remote] as a predictor variable. Results were not significant and did not change results presented in Table 3 and were, therefore, not included.

**Table 3:** Results of multinomial logistic regression using impact (decreased, no change, increased) of pandemic on capacity to provide direct services as dependent variable and gender, funding type, and primary workforce role as predictor variables

	*B*	*e*^*B*^	SE	Wald	*p*
Increased capacity vs no change					
Gender^a^	0.10	1.10	0.23	0.18	0.67
Mixed public/private funding^b^	**0.88**	**2.41**	**0.23**	**14.57**	**< 0.001**
Private funding^b^	**1.17**	**3.21**	**0.22**	**28.72**	**< 0.001**
Addiction counsellors^c^	0.08	1.08	0.36	0.05	0.83
Unregulated MHT^c^	− 0.48	0.62	0.26	3.30	0.07
Psychologist^c^	− **1.27**	**0.28**	**0.39**	**10.63**	**0.001**
Nurse^c^	− **0.84**	**0.43**	**0.29**	**8.08**	**0.004**
RSW^c^	− 0.44	0.65	0.24	3.34	0.07
Decreased capacity vs no change					
Gender^a^	− 0.26	0.77	0.23	1.22	0.27
Mixed public/private funding^b^	0.16	1.17	0.21	0.54	0.46
Private funding^b^	− 0.20	0.82	0.21	0.89	0.35
Addiction counsellors^c^	0.31	1.36	0.33	0.86	0.35
Unregulated MHT^c^	− 0.47	0.63	0.27	3.13	0.08
Psychologist^c^	− 0.44	0.65	0.33	1.75	0.19
Nurse^c^	− 0.45	0.64	0.26	2.96	0.09
RSW^c^	− 0.22	0.80	0.23	0.92	0.34

(*N* = 1044; 158 men, 886 women; 251 mixed, 313 private, 480 public; 97 addiction counsellors, 161 unregulated mental health therapist, 65 psychologists, 155 nurses, 265 registered social workers; 438 decreased, 251 no change, 355 increased). Bold highlights significant results. MHT = mental health therapist. RSW = registered social worker

^a^Women used as the reference group. Regression coefficient represents difference between men and women. Number of participants who indicated non-binary were too small to include in the analyses. ^b^Public funding used as the reference group. Mixed refers to a combination of public and private funding. Private funding refers to payments received from benefit programs, donations and endowments, or direct from clients. ^c^Regulated mental health therapist used as the reference group

### Reasons for change in MHSUH service provider capacity

Survey responses to the checklists of potential reasons for decreased or increased capacity are shown in Tables 4 and 5. The most common reasons for decreased capacity included lockdown and physical distancing measures, clients or patients lacking access to or comfort with virtual care, and additional COVID-19 protocols. The most common reasons for increased capacity included use of virtual care and more people having problems relevant to the participant’s skills. Survey responses to the checklist of potential factors that would enable participants to provide additional services that they were not currently providing are shown in Table 6.

**Table 4:** Reasons for decreased availability or ability to provide services

Reason for decrease		% of Sample (*n* = 1526)	% of Women (*n* = 1197)	% of Men (*n* = 239)
1	Lockdown or social/physical distancing measures	31.8	32.6	27.6
2	People/clients/patients lack access to or comfort with virtual care	26.2	26.6	23.8
3	Additional COVID-19 protocols slow down the rate of service provision	20.2	21.3	16.7
4	Additional personal/familial responsibilities	9.4	9.5	8.4
5	I am concerned about catching COVID-19	8.4	8.5	6.3
6	My own mental health and/or substance use concerns	7.7	7.7	5.4
7	I have had to take on tasks from others who are redeployed elsewhere	6.2	6.3	6.7
8	Lack of access to virtual care infrastructure (e.g., broadband, data, IT support)	5.1	5.0	5.9
9	I, or my employer, have decided to increase my time on other activities	3.9	4.2	2.5
10	I have personally been quarantined due to COVID-19 exposure	3.7	3.5	3.8
11	I require additional training in virtual service delivery	3.0	2.6	4.2
12	Limited access to PPE	1.8	1.8	2.1
13	I have caught COVID-19	0.9	1.2	0.0
14	Fewer people have problems relevant to my skills (e.g., grief, trauma)	0.9	0.8	1.7

Percentages based on number of participants who responded to the previous item asking if the pandemic had decreased, not changed, or increased their availability and/or ability to provide services. The *n* for the sample does not equal the sum of the ns for women and men because the *n* for the sample includes participants who chose another response option for gender (e.g., gender non-binary) or skipped the gender item. Percentages for men and women did not differ significantly based on Pearson's Chi-squared test of independence

**Table 5:** Reasons for increased availability or ability to provide services

Reason for increase		% of Sample (*n* = 1526)	% of Women (*n* = 1197)	% of Men (*n* = 239)
1	Use of virtual care	22.9	22.7	23.0
2	More people have problems relevant to my skills (e.g., grief, trauma)	13.4	13.3	14.2
3	Decided to provide more services on a voluntary basis	8.8	8.1	11.7
4	I receive referrals more quickly	8.5	8.4	10.0
5	New funding has been made available	5.4	**4.7**	**8.8**
6	Reduced administrative barriers	5.2	**4.0**	**10.9**
7	I, or my employer, have decided to decrease my time on other activities in order to provide more MHSUH services	4.8	4.5	6.3
8	I am able to provide more services outside my province/territory because of reduced regulatory barriers	3.7	**2.8**	**7.9**

Percentages based on number of participants who responded to the previous item asking if the pandemic had decreased, not changed, or increased their availability and/or ability to provide services. The *n* for the sample does not equal the sum of the ns for women and men because the *n* for the sample includes participants who chose another response option for gender (e.g., gender non-binary) or skipped the gender item. Percentages in bold indicate differences between men and women were significant at *p* < 0.01 based on Pearson’s Chi-squared test of independence

**Table 6:** Factors that would enable starting to provide additional services

What would enable starting to provide additional services?		% of sample (*n* = 821)	% of women (*n* = 639)	% of men (*n* = 147)
1	If there was funding/If you were paid to provide those services	37.8	38.7	34.7
2	If you could provide care virtually	21.9	21.9	23.8
3	If you were provided with additional training, including refresher training	22.0	21.0	26.5
4	If regulatory barriers were removed	23.5	23.5	22.4
5	If your organization enabled you to practice to optimal scope	24.2	23.9	27.2
6	If you were provided with additional coping resources and supports	26.8	27.9	21.8

Values for* n* are the number of participants who indicated in a previous question that they could start to provide additional services that they were not currently providing. Percentages for men and women did not differ significantly based on Pearson's Chi-squared test of independence

### Thematic analysis of key informant interviews

Results from the survey data analysis offered insight into changes to workforce capacity during the pandemic from the perspective of service providers by asking about changes to their practice and their availability and ability to provide services, as well as reasons for these changes. Given the dearth of empirical research in this area and the urgency for a robust workforce with adequate capacity to meet the MHSUH needs of Canadians, we also considered it important to explore issues affecting MHSUH workforce capacity from the perspective of key informants who were in a position to offer a broad view of the workforce and obstacles to workforce planning.

Interviews with the 13 key informants were transcribed verbatim and deductively coded in NVivo. We developed an initial coding framework based on the results of our literature review and preliminary findings of the survey. Coded data were thematically analyzed to identify key concepts raised in the interviews around the impact of the pandemic on workforce capacity. Coding and themes were refined over the course of consultations with the advisory committee and continued discussion amongst the research team, resulting in three interconnecting themes: the differential impact of public health measures, long-term effects of pandemic work conditions, and critical gaps in MHSUH workforce data.

### Differential impact of public health measures

The first broad theme constructed using thematic analysis involved the varying impacts of public health measures on MHSUH service providers. During the initial wave of the pandemic, the massive switch to virtual services because of physical distancing and lockdown measures was more complicated for some providers because they required training to use the necessary technology and lacked resources to provide services virtually (e.g., high-quality internet access, computers equipped with web cameras, technical support, private space in their homes to ensure confidentiality). Some jurisdictions in the publicly funded health care system were quicker than others to set up systems to pay providers (e.g., physicians, psychiatrists) and to adopt the technologies required for secure communications amongst health care providers and their clients; however, even within the same jurisdiction, some service settings (e.g., hospitals, community-based clinics) were faster than others to set up virtual services, with different settings offering different types of virtual services (e.g., telephone vs video-conferencing).

Despite the many challenges to adapting services for virtual delivery, service providers were driven by their dedication and concern for their clients to continue offering MHSUH services as soon as possible. As stated by one key informant from the peer support sector who spoke about their motivation to offer virtual programming as soon as possible after the announcement of lockdown measures: “Because I know what it is like to live with a mental health condition and to suddenly be alone and afraid and terrified. I knew that we as a program had to provide services” (Interview #1). The same key informant went on to explain, however, that some types of MHSUH services, including many peer support services provided in the community and in group formats, were more difficult to adapt to a virtual format.

Peer support is a community-based program. So we don’t meet in offices and stuff like that, we’re out in the community. So that made it a really huge challenge, because it would depend on the goal of the peer as to what the role of the peer support worker would be. For example, if they were going to the gym, well, no more, if they were going to recreational activities, no, cancelled, closed. If they were going to education things cancelled, closed. So all those things were impacted. (Interview #1)

As the pandemic moved into subsequent waves, the MHSUH workforce continued to face varying challenges. Key informants indicated that adequate staffing of in-person MHSUH services was an important concern. Unanticipated absences increased for numerous reasons, such as disruptions in childcare and self-isolation requirements for service providers when they or their family members had been in contact with or had symptoms of COVID-19. There were also increases in early retirements for older service providers who were at higher risk of experiencing severe COVID symptoms, and alternate working arrangements for other high-risk service providers.

Service providers delivering in-person services were also drastically affected by infection prevention protocols, which changed again and again as new information about the virus emerged. Key informants indicated that most in-patient and residential settings in hospitals and community-based organizations were not physically designed for infection control and lacked the necessary spaces for physical distancing (e.g., bathrooms for individual patients, separate spaces for simultaneous intake of new residents), which was especially challenging in acute situations involving patients experiencing psychosis or withdrawal symptoms. Isolation requirements for patients who were COVID-positive or waiting for test results also made it difficult to provide out-patient services, particularly in the homelessness service sector for patients who had no place to self-isolate.

Key informants also highlighted the differential impact of public health measures on unregulated compared to regulated occupations. Although the primary purpose of regulating health professionals is to protect the public from unsafe practices, key informants explained that there is "a divide and a power dynamic between regulated and unregulated roles in the field" (Interview #2) because regulation affects funding and resource allocation and contributes to recognition by governments and the general public as a valid source of MHSUH services. Unregulated peer support and community-based services in substance use and addiction were particularly affected as many of these services were not classified as essential during the pandemic and therefore struggled to access important resources such as personal protective equipment and to remain available during strict lockdown measures.

So most of the service providers in the substance use field are non-regulated. And a lot of services were not designated as essential, particularly community-based services and services being delivered by peers. So there was a lot of frustration in terms of ability to reach clients, ability to access personal protective equipment in order to keep themselves and the clients safe. (Interview #2)

Finally, there was strong agreement that public health measures during the pandemic had magnified existing inequities accessing services for particular populations. Lower quality internet service, which is more typical in remote and rural communities, made it more difficult for people living in these areas to access services, and travel restrictions meant that service providers who typically flew into remote communities were not able to do so. This combination of lower quality internet and travel restrictions had a significant impact on remote Indigenous communities in particular because these communities often rely on MHSUH service providers who travel into these communities.

So in particular, like Indigenous communities were significantly impacted. I know a lot of the staff psychiatrists who normally fly into those communities initially, were not able to travel there. And while there was an attempt to provide the care, like virtually, the internet infrastructure is very much sub-optimal in those communities. And so that, you know, unfortunately, it led as well once again to patients eventually having to be transported out of their communities down south to receive services. And that comes with a whole host of its own challenges. (Interview #4)

Inequities also increased for many individuals attempting to access substance use health or homelessness services. In addition to the difficulties faced by numerous unregulated service providers in these sectors who were not recognized as essential, many individuals in need of substance use health or homelessness services lacked the resources to access virtual services and to satisfy COVID-19 protocols (e.g., proof of negative COVID-19 test) required to physically enter facilities in order to access in-person services. Many did not own or have access to the technology (e.g., phones, computers) needed for virtual appointments with service providers, and even finding a safe space for such an appointment could be difficult. Other clients were not comfortable accessing services delivered in a virtual format.

That said, there were certain clients that didn’t want to switch to virtual care at all, they simply just dropped services completely, which is a whole other sort of black hole of clients that we lost. But I would imagine that those that are dealing with people who are marginalized, people in remote areas, homeless, street entrenched youth, those were the ones that were kind of really screaming out at the beginning of the pandemic, like you’re leaving this population behind if we switch to virtual care, because they really can’t jump on board the way certain other people can. (Interview #2).

### Long-term effects of pandemic work conditions

Key informants expressed concern about the long-term outlook for MHSUH provider capacity, particularly in the public sector, because of the potential impact of accumulating stress and burnout on workforce capacity because of increased need for sick leave, permanent departures from the workforce, turnover, and reduced morale. In addition to coping with changes to the way they work and staffing shortages, service providers have faced additional stressors related to the pandemic. For example, front-line workers have limited their contact with friends and family out of fear of transmitting COVID-19 and have consequently felt isolated themselves and worried about their caregiving responsibilities for children and older family members. There have also been higher levels of strain on MHSUH staff who were called on to support other healthcare workers involved in crisis situations and on providers who felt they were not providing the needed level of care to their clients and patients. Furthermore, the pandemic continued for longer than initially expected, and high levels of stress in the first wave of the pandemic continued to accumulate through subsequent waves.

And I think the impact on staff is just that the pandemic now is beginning to wear on people’s energy levels and their ability to continue to do their work in a really positive way. I think it’s lasted longer than anybody anticipated. And so, we’re starting to see some signs of burnout in some of our staff, most definitely, and having to try and troubleshoot around that and protect against that. (Interview #11)

The negative effects on the well-being of front-line MHSUH providers working in the homelessness and substance use health service sectors is of particular concern given that work conditions in these sectors have become stressful to the point that providers are leaving front-line positions. Key informants indicated that individuals who are homeless or experiencing withdrawal are often dealing with problems that are more urgent to them, such as finding a safe place to sleep, compared to following COVID-19 protocols, and front-line workers have had to take on the added risk and fear of catching COVID-19 in order to provide in-person services to these individuals. A key informant managing a community-based residential program described how their entire team was new because all the full-time workers in the program had resigned since the start of the pandemic for various reasons, such as concerns about catching COVID-19 and potentially taking it home to their families, as well as the challenges of providing front-line care during the pandemic:

Some [front-line workers], you know, could do it [provide front-line services] without the pandemic. Then you put the pandemic on top of that, and I think what happened is it proved to be too much. Because you have your own worries, your own concerns, and then you’re, you know, you're taking care of people, that the pandemic doesn’t really matter to them. But we found that, you know what, that COVID-19 for residents were the last thing on their list. It was going through withdrawal, their addiction, trying to find treatment, trying to find next steps and get stable. So I think for folks that already were maybe not so comfortable in the front line, the pandemic proved to be just that added push, that you know what, this isn’t for me. I need to do something where I feel I have more control, I need to do something where I'm not working with people that are in immediate crisis. (Interview #12)

Key informants also discussed how the substance use sector of the workforce was already experiencing burnout and grief prior to the pandemic because of the opioid crisis, which has worsened during the pandemic, and conveyed how seeing the vast number of resources allocated to the COVID-19 pandemic has contributed to further demoralization of a workforce that has been diligently working to prevent increasing numbers of overdoses and deaths.

We have to remember that these practitioners are still experiencing, you know, the opioid epidemic and overdosing epidemic. We hear a lot, you know, anecdotally, when we’re speaking to our peer support workers, of really traumatic issues. That they're seeing, you know, a lot of death in their communities. (Interview #2)

Finally, stressful working conditions during the pandemic are causing a shift to the private sector for MHSUH service providers. Key informants described the worries associated with delivering in-person services (e.g., fear of catching COVID-19 and spreading to family members) and the added stress of following infection prevention protocols, which have been continuously changing since the start of the pandemic. For those with the necessary credentials and skill sets, moving to a private sector position or opening a private practice to provide services that can be delivered virtually or to have more control over working conditions is one means of coping with the accumulating stress and burnout of the pandemic.

And I think COVID will have accelerated that shift that was kind of already there, in some ways, but a shift to private practice for social workers that have the skill set. So they may have been in public, or they have been, maybe a counselor and in a public system, and then, and then in this system, either moved into private, or they were already had their foot in private, and they just went full in private, and those that were already in private, they shifted to full electronic, or e-services. (Interview #8)

### Critical gaps in MHSUH workforce data

The view that critical gaps in data made it difficult to accurately assess the impact of the pandemic on the MHSUH workforce and to plan for future needs was a prevalent theme in our interviews. Key informants explained that the increased need for services during the pandemic and the expectation of continued increased need over the long-term highlighted the importance of data for workforce planning but that even basic information (e.g., number and type of service providers by region, sociodemographic data) about the current state of the workforce was nonexistent. There was also agreement about the need for more detailed information such as data on skill sets, well-being of service providers, anticipated graduations from training programs, and intentions to leave the MHSUH workforce by retirement or other means.

Key informants indicated that the workforce data that do exist are fragmented, with varying degrees of data collection and quality depending on the occupation and service sector. The main source of information used by many decision-makers is data from surveys conducted by various stakeholders across the country (e.g., governments, insurance companies, professional associations, unions, regulatory bodies). These surveys typically target a subset of the MHSUH workforce, such as members of a particular profession, rather than the entire workforce, and sampling methods result in data that are not representative of the particular group of service providers (e.g., psychotherapists, social workers, nurses) being surveyed. Moreover, the content of survey data depends on the interests of the organization conducting the survey, and data are not frequently shared between organizations or different levels of government.

Data are even more limited for occupations that are unregulated and uncertified. Key informants described difficulties collecting data from service providers in these occupations because these occupations do not have regulatory or certifying bodies that keep registration records with contact information. This is frequently the case for providers in the substance use health services sector. One key informant described how data are inadequate in general for mental health service providers but is particularly lacking in the substance use health field:

We know so little as to what’s going on in terms of numbers, scope, responsibility, workload. It's haphazard, it isn’t collected in any standardized way provincially. It's done regionally. And even then it’s very uneven across the country. I mean, we know really, we know so little about the substance use, I mean, when I think of psychology and some of the other mental health pieces, we know kind of bits and pieces, and some are better than others. But on the substance use side, we're at a whole different level in terms of lack of statistical data capture in a meaningful way. I mean, it’s just a huge chasm. (Interview #7)

Another key informant explained the difficulty with knowing the impact of the COVID-19 pandemic on the substance use health workforce:

The challenge in this field is we didn’t have a good understanding of what the capacity was before COVID, which makes it extremely difficult to then anticipate how much ground we have to make up post-COVID in order to not just mitigate the impact on reduced capacity of the existing workforce, but also scale up to meet anticipated increase in demand in the population. So in my perfect rainbow world, I would have data about the existing capacity of the substance use workforce, including both regulated and unregulated providers, so that we could make those estimates in terms of what’s needed to close the gaps. (Interview #2)

## Discussion

The goal of this research was to systematically examine the impact of the COVID-19 pandemic on the capacity of the MHSUH workforce across Canada. Findings indicate that, although capacity fluctuated somewhat over the course of the pandemic as adjustments were made to cope with the initial shock of emergency measures and subsequent waves of the pandemic, a substantial portion of the workforce decreased capacity, but that capacity of other service providers remained the same or increased. Findings also indicate that both the private and public sectors have faced substantial challenges but that privately funded service providers were more likely to have increased capacity. This is particularly concerning from an equity perspective for lower-income Canadians if private sector growth comes at the expense of an overburdened public sector.

Results from the study align with existing findings that the COVID-19 pandemic has had an enormous impact on MHSUH services [e.g., 3, 4] and highlight that COVID-19 public health measures have had varied effects on the capacity of MHSUH service providers. Not surprisingly, survey participants confirmed a massive shift from face-to-face to virtual care because of lockdown and physical distancing protocols, and key informants described a tumultuous period at the beginning of the pandemic when the workforce began adapting care to a virtual setting. Service providers had varying levels of experience and comfort using virtual technologies, as well as varying levels of resources such as high-quality internet access, technical support, and training, which made the transition more challenging for some. We also found that in-person services that did continue were severely affected by frequently changing COVID-19 protocols implemented to limit viral transmission, particularly in community-based and hospital in-patient and residential settings, which lacked the infrastructure necessary for infection control.

Our findings confirm that regulation played a major role in workforce capacity because of the difficulties faced by unregulated service providers (e.g., mental health therapists and counsellors, addictions counsellors, peer support) such as not being covered by employee-sponsored health insurance, not being designated as essential in times of crisis, and lack of access to personal protective equipment. Those working in unregulated occupations also struggle to be viewed as legitimate MHSUH service providers [21], and many work for low pay and no benefits with high job insecurity [22], which is associated with increased burnout and poor psychological well-being [23].

Increased gender inequality in caregiving and work responsibilities [24, 25] is reflected in the larger percentage of women in our survey sample who indicated their capacity to provide services during the pandemic has decreased and the larger percentage of men who indicated their capacity has increased. It is somewhat surprising that the effect of gender in the logistic regression analyses was not larger. We speculate that the especially stressful work conditions for all healthcare workers during the pandemic [26, 27], the overlap of gender and publicly funded participants (i.e., the higher percentage of women in the public sector compared to the private sector), and the unbalanced gender composition of our sample statistically masked some of the existing gender differences in MHSUH workforce capacity. Moreover, it is likely that healthcare workers with the highest burden did not have time to participate in an online survey, which is one of the limitations of our methodology.

The current research also highlights that, in addition to inequities faced by MHSUH service providers, barriers to delivering and accessing virtual services during the pandemic have magnified existing inequities accessing services. For example, low-income groups and individuals experiencing homelessness often do not have access to the necessary technology or a suitable location that is safe and private for a virtual appointment. The complexities of addictions treatment mean that many services in this sector are less suitable for virtual delivery, and some services that could not be provided virtually were discontinued. In addition, regions with lower quality internet infrastructure, including many remote and Indigenous communities, are at a disadvantage not only because accessing MHSUH services virtually is more difficult compared to urban areas but also because travel restrictions and COVID-19 protocols that limited the number of locations where a service provider can work decreased the availability of service providers to provide coverage in these areas.

### Limitations

This research makes an important contribution to knowledge about the MHSUH workforce; however, there are several important limitations to consider. The cross-sectional survey design does not capture fluctuations in capacity across the stages of the pandemic or allow for causal conclusions. It is also important to note that the retrospective design of both the survey and the interviews may have introduced recall bias into responses to questions about pre-pandemic work characteristics [28]. Moreover, given that participants were not randomly selected for the survey, the sample is not representative of the MHSUH workforce. It is likely that members of the MHSUH workforce who were experiencing the highest demands did not have the time or energy to participate in an online survey, which is one possible explanation for the non-significant results of the multinomial logistic regression comparing decreased capacity to no change in capacity. Given the paucity of data on the Canadian MHSUH workforce, it is difficult to comment further on differences between the survey sample and the population, but we urge caution in terms of generalizability of our findings.

The combination of surveying MHSUH service providers and interviewing key informants is a strength of our research, but it is important to acknowledge that our findings are limited in terms of the impact of the COVID-19 pandemic on sub-groups of the workforce with intersecting social identities. Although recruitment efforts were made to include participants with diverse sociodemographic characteristics, the survey sample did not have large enough proportions for a more in-depth sub-groups analysis. Future research with a specific focus on intersectionality will be essential to understand how the disproportionate impact of the pandemic on women, minorities, and Indigenous groups has affected Canada's ability to provide culturally appropriate MHSUH services, which was already a major concern prior to the pandemic.

### Implications

The current research has important implications for MHSUH workforce policy. The COVID-19 pandemic has magnified existing inequities accessing MSHUSH services in Canada, and policy to address these inequities must be prioritized. First, the overarching implication of our research is that systematic collection of standardized data describing the MHSUH workforce at the national level is a fundamental requirement. Participants in our survey worked in over 20 different occupations; yet, only psychologists, physicians, nurses, social workers, and occupational therapists are included in health workforce data collected by the Canadian Institute for Health Information (CIHI) [29], a pan-Canadian health organization funded by the federal, provincial and territorial governments to provide data and analyses on Canada's health systems. To build and maintain a stable MHSUH workforce capable of meeting population needs, workforce planning cannot continue to rely on survey data that are non-representative and inadequate for making comparisons over time. Decision-makers need access to high-quality data from all occupations delivering MHSUH services nationwide, including data on training programs and planned retirements.

Second, increased public funding for MHSUH services is urgently needed. Canadians without access to employer-sponsored insurance coverage or the money to pay out-of-pocket face financial barriers to accessing services provided by the private sector. In addition, community-based and hospital settings require additional public investment to meet the unique infrastructure challenges for infection control in residential and in-patient settings.

Finally, considering our findings that stressful work conditions during the pandemic pushed MHSUH service providers out of front-line positions and contributed to turnover and increased sick leave, it is more important than ever to focus on regulating Canada's MHSUH workforce. Expanding regulation of MHSUH occupations would increase workforce capacity by facilitating inclusion in health workforce planning, improving work conditions, pay, and benefits to support a precarious workforce (e.g., homelessness and addictions service sectors), and increasing the likelihood of being designated as essential workers that require access to resources (e.g., PPE, funding) in the event of future crises. Successes in advocacy for regulated service providers such as registered social workers and counsellors to be covered by private insurance for mental health-related counselling services during the pandemic have clearly demonstrated the advantages of working in a regulated MHSUH occupation. Regulation would also serve as quality control with minimum entry-to-profession requirements and would provide a viable means of collecting standardized MHSUH workforce data.

## Conclusion

The COVID-19 pandemic contributed to alarming increases in need for MHSUH services, highlighting the need for better health workforce planning in Canada. At the same time, the pandemic has drastically changed how MHSUH services are provided, which has contributed to changes in capacity of the MHSUH workforce to provide these services. Notably, the capacity of the private sector increased compared to the public sector, and the massive shift to virtual care created barriers for those without the technology or financial means to access virtual services. This raises important equity concerns with respect to provision and access of MHSUH services for vulnerable groups. Finally, distressing work conditions that have contributed to elevated stress and burnout are particularly concerning for the long-term outlook for workforce capacity, and implementation of systematic, nationwide data collection that includes all MHSUH occupations is urgently needed for workforce planning.

## Supplementary Information

**Additional file 1.** Supplementary Data: Hours and clients per week before and during the COVID-19 pandemic by primary workforce roleand gender.

## Data Availability

The datasets used and/or analyzed are available from the corresponding author on reasonable request.

## Abbreviations

CIHI: Canadian Institute for Health Information
MHSUH: Mental health and substance use health
