# Supplementary material for: The impact of COVID-19 on the mental health and substance use health (MHSUH) workforce in Canada: a mixed methods study
Source: Hum Resour Health. 2023 Feb 8;21:9. doi: 10.1186/s12960-023-00797-6 (PMC9907177; doi:10.1186/s12960-023-00797-6)
Supplement: Supplementary file 1 — Additional file 1. Supplementary Data: Hours and clients per week before and during the COVID-19 pandemic by primary workforce roleand gender. [file 12960_2023_797_MOESM1_ESM.docx]

Table S1. Hours and Clients Per Week Before and During the Pandemic by Primary Workforce Role

|  |  | Before | | | |  | During | | | | |  | |  | |  | |  | |  | 95% CI | |
| --- | --- | --- | --- | --- | --- | --- | --- | --- | --- | --- | --- | --- | --- | --- | --- | --- | --- | --- | --- | --- | --- | --- |
| Variable | *N* | *M* | *Mdn* | *IQR* | *SD* |  | *M* | *Mdn* | *IQR* | *SD* |  | | *ΔM* | | *t* | | *p* | |  | | LL | UL |
| Hours per week |  |  |  |  |  |  |  |  |  |  |  | |  | |  | |  | |  | |  |  |
| Addiction Counsellors | 102 | 30.4 | 35.0 | 16.0 | 14.3 |  | 30.4 | 35.0 | 15.9 | 13.9 |  | | 0.0 | | 0.05 | | .96 | |  | | -2.1 | 2.3 |
| Unregulated MHT^a^ | 172 | 27.3 | 25.0 | 22.0 | 18.3 |  | 25.5 | 25.0 | 21.2 | 14.7 |  | | -1.7 | | -1.71 | | .09 | |  | | -3.7 | 0.3 |
| Psychologist | 69 | 29.1 | 30.0 | 20.0 | 13.3 |  | 29.2 | 30.0 | 19.0 | 13.5 |  | | 0.1 | | 0.06 | | .95 | |  | | -2.3 | 2.4 |
| Nurse | 167 | 29.7 | 35.0 | 18.0 | 16.4 |  | 30.0 | 35.0 | 24.0 | 14.9 |  | | 0.3 | | 0.43 | | .67 | |  | | -1.1 | 1.8 |
| **RSW** | **280** | **27.8** | **30.0** | **18.0** | **11.3** |  | **29.9** | **34.0** | **20.0** | **13.5** |  | | **2.0** | | **4.12** | | **<.001** | |  | | **1.1** | **3.0** |
| **Regulated MHT^a^** | **325** | **25.5** | **25.0** | **20.0** | **12.6** |  | **27.9** | **28.0** | **20.5** | **14.3** |  | | **2.4** | | **4.51** | | **<.001** | |  | | **1.3** | **3.4** |
| Clients per week |  |  |  |  |  |  |  |  |  |  |  | |  | |  | |  | |  | |  |  |
| Addiction Counsellors | 102 | 20.8 | 16.0 | 15.0 | 16.8 |  | 21.4 | 20.0 | 17.8 | 16.1 |  | | 0.6 | | 0.47 | | .64 | |  | | -2.0 | 3.2 |
| Unregulated MHT^a^ | 169 | 19.3 | 20.0 | 13.0 | 12.2 |  | 19.9 | 18.0 | 15.0 | 12.5 |  | | 0.6 | | 0.87 | | .39 | |  | | -0.8 | 2.0 |
| Psychologist | 68 | 18.2 | 15.3 | 15.0 | 10.7 |  | 19.3 | 18.0 | 14.5 | 11.2 |  | | 1.1 | | 1.56 | | .12 | |  | | -0.3 | 2.6 |
| Nurse | 168 | 27.2 | 15.0 | 22.0 | 51.5 |  | 32.4 | 15.0 | 26.8 | 70.4 |  | | 5.3 | | 1.29 | | .20 | |  | | -2.8 | 13.4 |
| **RSW** | **276** | **20.7** | **17.0** | **15.0** | **18.0** |  | **24.1** | **20.0** | **18.00** | **20.2** |  | | **3.3** | | **5.05** | | **<.001** | |  | | **2.0** | **4.7** |
| **Regulated MHT^a^** | **323** | **18.1** | **18.0** | **10.0** | **9.8** |  | **20.8** | **20.0** | **12.0** | **11.8** |  | | **2.6** | | **5.85** | | **<.001** | |  | | **1.8** | **3.5** |

*Notes*. IQR = interquartile range. MHT = mental health therapist. RSW = registered social worker.

^a^ Regulated refers to participants who indicated being a member of a regulatory body.

Table S2. Hours and Clients Per Week Before and During the Pandemic by Gender

|  |  | Before | | | |  | During | | | | |  | |  | |  |  |  | 95% CI | |
| --- | --- | --- | --- | --- | --- | --- | --- | --- | --- | --- | --- | --- | --- | --- | --- | --- | --- | --- | --- | --- |
| Variable | *N* | *M* | *Mdn* | *IQR* | *SD* |  | *M* | *Mdn* | *IQR* | *SD* |  | | *ΔM* | | *t* | | *p* |  | LL | UL |
| Hours per week |  |  |  |  |  |  |  |  |  |  |  | |  | |  | |  |  |  |  |
| **Woman** | **1119** | **27.5** | **30.0** | **20.0** | **13.5** |  | **28.7** | **30.0** | **17.5** | **14.3** |  | | **1.2** | | **4.42** | | **<.001** |  | **0.7** | **1.8** |
| Man | 233 | 30.0 | 30.0 | 25.0 | 20.0 |  | 28.5 | 30.0 | 28.0 | 17.1 |  | | -1.5 | | -1.74 | | .09 |  | -3.2 | 0.2 |
| Clients per week |  |  |  |  |  |  |  |  |  |  |  | |  | |  | |  |  |  |  |
| **Woman** | **1110** | **21.2** | **16.0** | **15.0** | **30.5** |  | **22.9** | **20.0** | **15.3** | **26.2** |  | | **1.6** | | **4.40** | | **<.001** |  | **0.9** | **2.3** |
| Man | 230 | 24.0 | 20.0 | 20.0 | 19.5 |  | 27.2 | 20.0 | 20.0 | 51.2 |  | | 3.1 | | 1.03 | | .31 |  | -2.9 | 9.1 |

*Notes*. IQR = interquartile range.
